# Aerial imagery dataset of lost oil wells

**DOI:** 10.1038/s41597-024-03820-0

**Published:** 2024-09-17

**Authors:** Anastasiia Kim, Teeratorn Kadeethum, Christine Downs, Hari S. Viswanathan, Daniel O’Malley

**Affiliations:** 1 Los Alamos National Laboratory, Los Alamos, New Mexico 87545 USA; 2 Sandia National Laboratory, Albuquerque, New Mexico 87185 USA

**Keywords:** Environmental impact, Climate-change mitigation

## Abstract

Orphaned wells are wells for which the operator is unknown or insolvent. The location of hundreds of thousands of these wells remain unknown in the United States alone. Cost-effective techniques are essential to locate orphaned wells to address environmental problems. In this paper, we present a dataset consisting of 120,948 aerial images of recently documented orphan wells. Each of these 512 × 512 images is paired with segmentation masks that indicate the presence or absence of such well. These images, sourced from the National Agriculture Imagery Program, cover the continental United States with spatial resolutions ranging from 30 centimeters to 1 meter. Additionally, we included negative examples by selecting locations uniformly across the United States. Accompanying metadata includes the IDs and spatial resolution of the original images, which are available for free through the United States Geological Survey, and the pixel coordinates of documented orphaned wells identified in these images. This dataset is intended to support the development of deep-learning models that can help locating undocumented orphan wells from such imagery, thereby blunting the environmental damage they do.

## Background & Summary

At least several hundred thousand oil and gas wells are improperly abandoned and do not have a solvent owner. These wells fall under the jurisdiction of governmental bodies and the public for management and plugging to mitigate their harmful environmental impact. A national effort, funded by the Bipartisan Infrastructure Law, has been initiated to remediate these wells, which are notably hazardous due to their propensity to contaminate groundwater, generate air pollution, and emit methane^1,2^. It was found that states recording the highest annual methane emissions are also those with the largest number of orphaned wells^3^. By April 2022, 123,318 orphaned wells were documented, constituting 3% of all estimated abandoned wells in the United States^2,4^. These contribute 5–6% of the total methane emissions, as estimated by the U.S. Environmental Protection Agency for all abandoned oil and gas wells^4^.

New capabilities for locating orphaned wells are a critical national need within the United States, as demonstrated by the $4.7 billion allocation from the U.S. federal government to plug and properly abandon these wells. Yet, optimizing the environmental benefits of such remediation requires closing existing data gaps. The term “documented orphaned well”, while denoting wells logged in state databases, varies in interpretation across states due to non-uniform documentation and verification processes. Such inconsistency, combined with data discrepancies, magnifies the challenge of building a unified national dataset on orphaned wells.

Boutot *et al*.^3^ extracted the most recent data from online state databases. Their analysis reveals a significant rise in the number of documented orphaned wells, from 81,857 in September 2021 to 123,318 in April 2022. The increase comes from the revision and updating of old databases with new well inventories, more field inspections, and the bankruptcy of oil and gas companies. Boutot *et al*.^3^ observed the pattern: new orphaned wells frequently appear in proximity to previously identified ones, particularly in historically oil-rich regions such as Pennsylvania, New York, and Ohio. This insight suggests that concentrating efforts in these regions could yield more undiscovered wells. However, many of these documented wells come with incomplete data — missing critical attributes like well type, production date, and depth. This is either due to state oversights or because the details reside in non-digitized archives, all records have been lost, or no records ever existed. After all, these wells go back as far as the 1850s when there was no expectation, let alone a legal requirement, to document an oil and gas well. Fluctuations in oil prices along with state regulatory and policy changes could be factors leading to wells becoming orphaned.

Estimates indicate hundreds of thousands of orphaned oil and gas wells are scattered across the United States. Utilizing data from recently located orphaned wells can help establish a cost-efficient framework for identifying and characterizing orphaned wells that have not yet been found.

This study focuses on using available orphaned well locations released by Boutot *et al*.^3^ to create a dataset from aerial images of these recently located wells, which can be used to locate more wells. Our dataset comprises 120,948 natural color images derived from the National Agriculture Imagery Program (NAIP) through the U.S. Geological Survey, the best freely available source for high-resolution aerial images. We cropped large-scale NAIP images to form a dataset of 58,882 images containing documented orphan wells and 62,066 images without wells. In addition to the images, segmentation masks have been generated to facilitate image-based analysis. Our intention is to make this dataset accessible to the machine learning community and to employ machine learning models specifically trained to segment, localize, or classify these images, thereby differentiating between areas that contain wells and those that do not.

## Methods

**Fig. 1:**
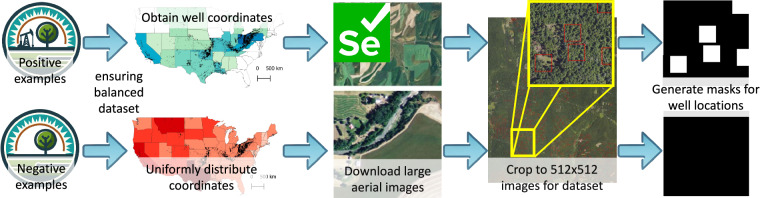
A flowchart outlining our approach for automatic data acquisition of NAIP aerial images from the USGS. We create a balanced dataset by acquiring an approximately 62,066 samples from both the Boutot well location dataset and a uniform distribution (about 120,948 images total). Then, we use the USGS API and Selenium to download large aerial images showing the relevant coordinates. Next, we extract smaller images (512-by-512 pixels) that cover the relevant coordinates within these large images. Finally, we generate masks that highlight well locations. The 512-by-512 pixel aerial images and their masks comprise our dataset.

We constructed this dataset to include the images and corresponding segmentation masks that indicate the presence or absence of wells. Our process involved selecting aerial imagery and geographical coordinates, downloading and further processing this imagery, generating segmentation masks, and recording the metadata for each image (Fig. 1).

### Locations of wells

The locations of documented orphaned wells were sourced from the “Documented Orphaned Oil and Gas Well” dataset spreadsheets released by Boutot *et al*.^3^. Two spreadsheets were released: one in 2021 containing 77,871 wells and another in 2022 containing 115,565 wells. We focused on the recently discovered wells, specifically those present in the 2022 dataset but not in the 2021 version, to focus on newly discovered wells. After eliminating duplicates, we found 25,674 wells present in both datasets, resulting in a total of 82,218 wells unique to the most recent dataset. Each well was characterized by its latitude, longitude, the state in which it was located, and a note indicating the accuracy of the well location. These well coordinate locations were used to produce positive images (i.e., ones with at least one well).

We note that our focus was on newly discovered wells, as they serve as the closest proxy to undocumented orphaned wells. There are potential differences in characteristics between older and newer wells; therefore, by concentrating on the newer wells, we aim to ensure that the trained models are more accurately attuned to the features of wells that have yet to be discovered.

To generate negative examples, we produced 100,000 random coordinates, bounded by the northernmost, southernmost, westernmost, and easternmost US coordinates. Some of these locations did not have NAIP images, either because they pointed to areas in water that are far from the shoreline or they were located in Mexico or Canada, resulting in a total of 62,066 negative images. We made sure to produce a sufficient number of negative examples to complement the positive ones. By performing an envelope calculation to estimate the occurrence of false positive wells from generated negative examples, we determined that the likelihood of such occurrence is less than 1%.

### Image acquisition

To access images, we first registered on the USGS platform and obtained an API (Application Programming Interface) key to be able to freely browse and download images from USGS servers. There are two ways to access USGS images: one is via their machine-to-machine API (https://m2m.cr.usgs.gov/api/docs/), and the other is by directly navigating and downloading from the Earth Explorer website (https://earthexplorer.usgs.gov/).

Our initial approach utilized the API. We inputted latitude and longitude coordinates and set the acquisition year to 2015 or later to acquire most recent images. The API returned URLs for downloading data, which remained valid for 48 hours after the initial download. In most cases the API returned multiple scenes, individual aerial images covering specific geographic areas at a particular point in time, each with its respective identification number, spatial resolution, acquisition date, and a display ID to locate the image on the Earth Explorer. We faced several challenges with the API, such as download rate limits imposed by the server, timeouts because of high server loads, and latency with “staging/preparing” URLs. These issues often meant that download links were not consistently generated on the first try, and specific images linked to the URLs were not immediately available for download. Therefore, we decided that API was not an effective approach for collecting the images. Instead, we used the API only for metadata collection. In particular, we used the open-source *m2m-api* (https://github.com/Fergui/m2m-api) which provides a more user-friendly implementation for making requests through the original API website. This allowed us to collect the following metadata: the list of multiple scenes and display IDs, years, and spatial resolutions for each well location. We then aggregated all scene identifiers available post-2015 for each location. In most cases, we were able to download the most recent images from 2022 with the best spatial resolution available. For certain locations, we were unable to acquire the most recent NAIP images with the highest spatial resolution. This was either because these images were not available for download as of August 2023 or because it was not possible to locate the wells on the tiles in cases when wells were located in the very corners of NAIP images. We note that while we prioritized recent images to improve the resolution, there is a tradeoff here. Older images may add value because they are less likely to lose visual information about the well to the ravages of time. Of course, they have lower resolution, which also reduces the information content – hence the tradeoff. Collecting older images is a potentially interesting path that we leave to future work.

With the scene IDs in hand, we used Selenium (https://www.selenium.dev), a web browser automation framework, to access and download the images from the Earth Explorer. Selenium was particularly useful because downloading from the Earth Explorer required simulating user interactions, such as searching with the scene ID and clicking the “Download” button. Selenium efficiently executed these actions iteratively for tens of thousands of images.

### Image processing

**Fig. 2:**
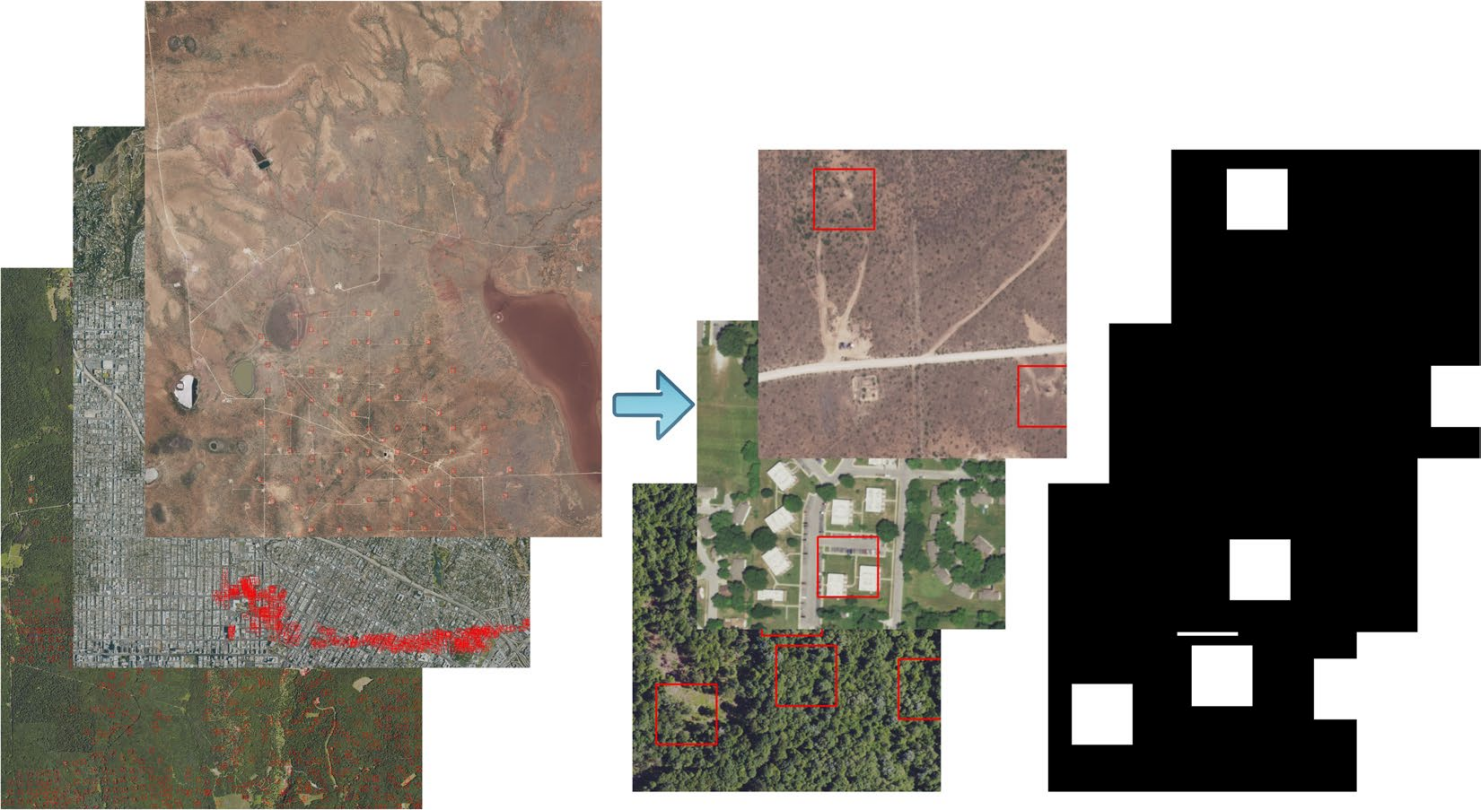
Generation of smaller tiles and corresponding masks from NAIP images. In some cases, more than one well is present in a single tile.

The size of NAIP images, in GeoTIFF format, varied significantly, ranging from 300MB to 2GB. A GeoTIFF is an enhanced version of the TIFF image format, specifically designed to store geospatial information. This means that, in addition to the visual image data, it embeds details about geographical coordinates (north, south, east, and west bounding coordinates), map projections, and real-world locations. During the downloading process, we cropped these large images into smaller images, tiles of size 512  × 512 pixels (Fig. 2). Since we had the locations of the documented wells, we projected latitude and longitude decimal degree coordinates of the wells to NAIP images coordinate reference system (NAD 83), and then identified the pixel locations of the wells on the images. This process resulted in 512  × 512 TIFF images with the single or multiple wells on them. All NAIP images we collected contained four color channels (RGB and near-infrared); during the image processing, we removed the near-infrared channel to obtain images in natural color as many pre-trained models (e.g., ResNet, VGG13, YOLO, U-net, etc.) were trained on datasets composed of RGB images (e.g., ImageNet, COCO, etc.) and thus expect inputs to be in the same format for optimal performance. Although these pre-trained models were mostly trained on standard camera images, rather than overhead imagery, one can leverage their foundation learning and fine-tune them with acquired aerial imagery.

**Fig. 3:**
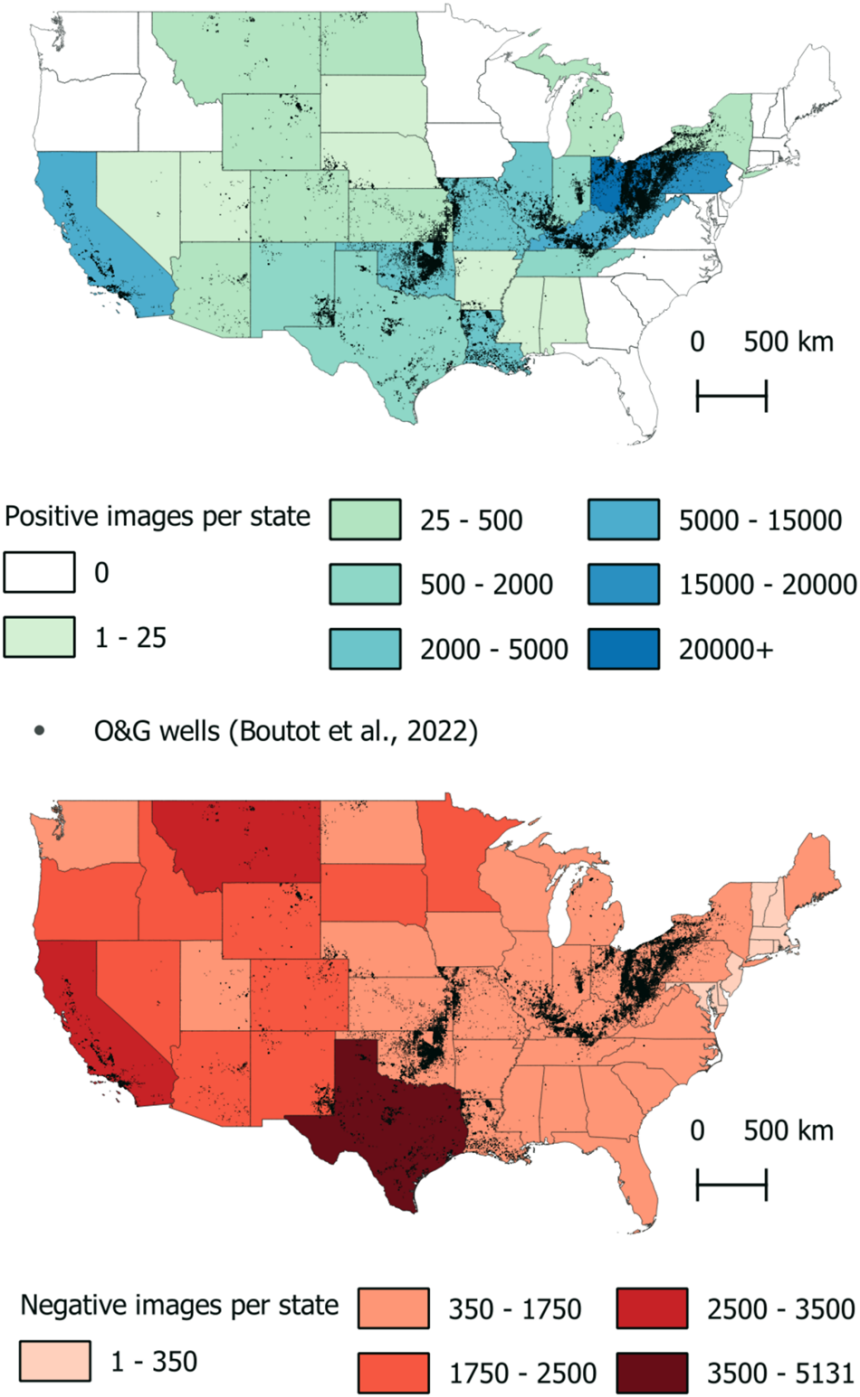
Documented orphaned well distribution across United States (Boutot *et al*.^3^) and number of positive and negative NAIP images by state. Note: Although the O&G well database includes 12 wells outside the continental United States (Alaska, not shown), there is no NAIP imagery from those locations.

We obtained 120,948 natural color images, each of 512 × 512 pixels, including 58,882 images containing orphan wells and 62,066 without wells (Fig. 3). Out of the total 82,218 well location coordinates, we were unable to acquire NAIP images for 125 locations due to the absence of NAIP imagery in Alaska and the placement of some coordinates in coastal waters where NAIP images are not available. The count of images containing wells was lower than the total number of wells of 82,218 due to the presence of 10,853 images with multiple wells. There were 184 images, each containing over 10 wells, accounting for 3,051 wells, with the majority located in California and Pennsylvania. States with the highest number of orphaned wells were Ohio (25%), Pennsylvania (20%), and Kentucky (16%), which together accounted for about 61% of the total number of documented wells.

For positive examples, a large proportion of the images in our dataset were sourced from 2022 (59%) and 2021 (38%). In terms of image spatial resolution, the dataset was primarily composed of images with the spatial resolution of 60 centimeters (~92%). Images with the resolution of 30 centimeters comprised about 5% of the dataset, and those with the resolution of 50 centimeters constituted approximately 2.5%. The rest were images at a 1-meter resolution, totaling only 7 images. For negative examples, the majority of the images were coming from 2022 (52%) and 2021 (43%) with the spatial resolution of either 60 centimeters (92%) or 30 centimeters (8%), which was closely aligned with the ratios for the set of positive images.

Since we cropped NAIP images into smaller tiles, some images, positive or negative, had black portions, particularly at the bottom or at the right edge, representing regions beyond the boundary of the actual imagery. Although it’s extremely rare, some NAIP images can be almost entirely black or blue, with only a small strip of land visible, resulting in predominantly black or blue tiles. This may occur in cases when the NAIP image is an edge image due to data corruption or if it contains large water bodies. We encountered such images in a few negative examples but chose to retain them in the dataset, as they will not affect model training performance (e.g., bodies of water are very likely to be true negatives). For the negative examples, locations were distributed uniformly, which resulted in smaller states like Rhode Island having the smallest number of images (24). Conversely, Texas, due to its larger size, had the largest number of images (5131). By spreading the negative examples uniformly across the continental US, models trained with the data will provide accurate performance in all areas.

Each 512  × 512 tile TIFF tile image was named using three numbers separated by underscores: the scene ID followed by a coordinate indicating the tile’s vertical position relative to the height of the entire image, and then followed by the tile’s horizontal position in relation to the image’s width. The coordinate system was established with its origin at the top left corner. This naming scheme ensured that each tile could be easily identified and located based on its unique position, aiding in the organization and retrieval of specific image sections.

### Image mask generation

For generating image masks with dimensions of 512  × 512 pixels, we produced two types of masks: positive and negative. In positive examples, the mask consisted of a predominantly black PNG image with a white square, each side measuring 100 pixels, corresponding to an area of 30-100 m^2^. This region was intended to be large enough to account for imprecise well location information – the well should be within the square the vast majority of the time. This square was positioned where the well was located on the image, serving as an identifier for the well. It should be noted that in cases when there were multiple wells on a tile, a white square was placed for each well, potentially leading to overlapping squares if the wells were closely positioned (Fig. 2). In contrast, negative examples featured an entirely black PNG image as the mask. This design distinction between the masks would aid in differentiating images that contain wells from those that do not. Each mask was named to match its corresponding 512  × 512 tile TIFF image.

### Metadata

We collected metadata for each well location, recording state where the well is, decimal coordinates of the well, spatial resolution, display and scene IDs of NAIP image, height and width of the NAIP image as well as its image bounding box coordinates, the filename of the corresponding 512  × 512 tile and pixel coordinates of the well on this tile. For many well locations, multiple NAIP images were available, we kept metadata of these “alternative” images to download later if necessary. For images not containing wells, i.e. negative examples, comparable metadata were preserved, albeit excluding information about “alternative” images.

### NAIP program

The NAIP provides free high-resolution aerial imagery of the U.S. since 2003 under U.S. public domain license^5^https://naip-usdaonline.hub.arcgis.com/. Initially capturing images at a 1-meter resolution during the agricultural “leaf-on” seasons, the standard was updated to a 0.6-meter resolution in 2018, with some coastal states even providing a 0.3-meter resolution. Additionally, after 2009 the repeat flight cycle was changed to no more than a 3-year cycle, ensuring more frequent updates in response to user needs. These images, spanning 3.75-minute longitude and latitude quadrants with added buffers, offer natural and near infra-red color options, and can include up to 10% cloud cover. Distributed by the USGS’s Earth Resources Observation and Science Center, the images come in GeoTIFF and JPEG2000 formats. These images are aligned with the Universal Transverse Mercator (UTM) coordinate system and referenced to the North American Datum of 1983 (NAD 83). The NAIP emphasizes accuracy, with post-2006 images tied to ground control points and rigorous testing to ensure a 95% confidence level in their precision. Some states have released more information about the quality and collection of NAIP data^6^. NAIP continuously makes progressive improvements in the quality of the imagery (see Table 1 in Maxwell *et al*.^7^ for details).

## Technical Validation

We ensured data quality by aligning WGS 84 well coordinates with NAD 83 UTM for NAIP images, allowing for precise well location identification. Mask accuracy was verified by visually inspecting approximately 100 images with wells in low-vegetation areas. We kept wells with inaccurate locations to later re-estimate them using the trained model.

**Fig. 4:**
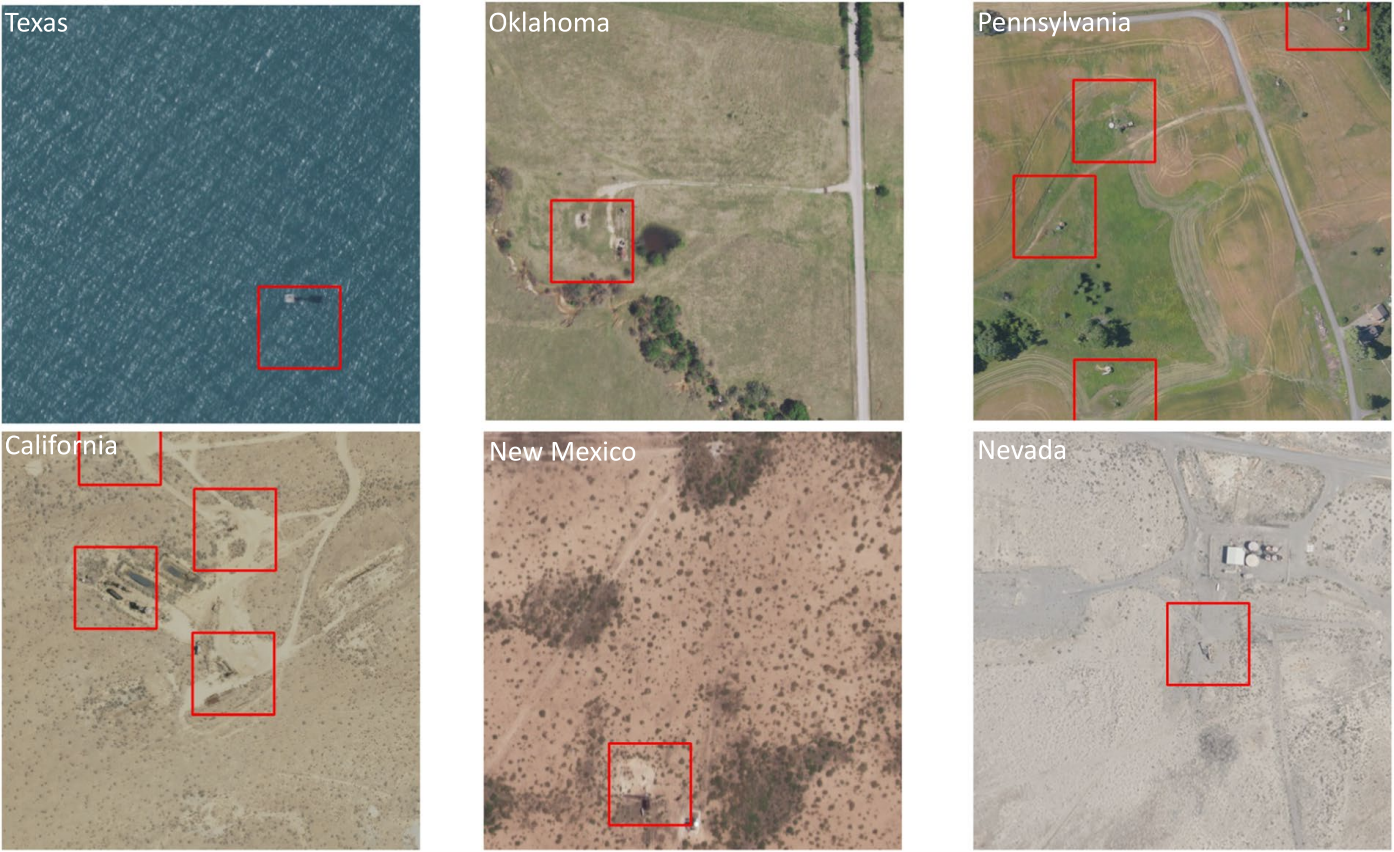
Wells visible to the human eye in low vegetation areas or in water bodies, surrounded by red squares. In some places multiple wells may be located in close proximity to each other.

The well locations were obtained from the spreadsheets released by Boutot *et al*.^3^. We accessed NAIP images from the Earth Explorer website directly using Selenium which ensured that the images were accessed in the exact manner any user would, navigating the website and fetching the desired NAIP imagery. This approach mimicked the natural behavior of a user, making it more likely to retrieve all the relevant and dynamically loaded content. Due to the well’s coordinates being based on the WGS 84 coordinate reference system (CRS) and the NAIP image being under the NAD 83 UTM CRS, we aligned the two different CRSs - WGS 84 for the well’s coordinates and NAD 83 UTM for the NAIP image. This allowed us to accurately find the location of the well on the NAIP imagery from decimal coordinates. This made the process of 512  × 512 tile identification and well location on the tile trustworthy. To validate the accuracy of mask generation, we generated red squares surrounding the well locations for rapid visual identification. We evaluated approximatelly 100 images, primarily in low-vegetation areas, to confirm the masks provide accurate representations of well locations (see Fig. 4 for some examples). Using low-vegetation areas makes it easier to visually identify the location of a well. In some instances, objects resembling wells were positioned several meters away from the provided coordinates. This discrepancy led us to enlarge the squares in the masks for the positive images, noting that the actual locations of some wells can deviate from the coordinates provided by Boutot *et al*.^3^. Our masks, with a dimension of 100 pixels square, were drawn to capture wells that might slightly deviate from their documented locations, occupying approximately 4% of the image in the majority of cases with a single well present. This size corresponds to an area of 30 to 100 meters squared on the ground, considering a spatial resolution of 0.3 or 1 meters per pixel. This large buffer was chosen after we visually inspected tens of images with low vegetation and determined that such a buffer was necessary to capture objects that resemble pumpjacks or well pads. We note that locating a well within  ~ 60 meters is sufficiently close to fly a drone with a magnetometer over the area and rapidly find a more precise location.

There were 529 wells for which the location was inaccurate, primarily located in Kansas, as documented in the spreadsheet data provided by Boutot *et al*.^3^. In particular, all well locations in Kansas (399 wells) were approximated on the basis of the geographical centroid of the section-township-range information provided by the oil and gas regulatory agency^3^. The other inaccuracies arise from low latitude or longitude precision, with these wells primarily located in Pennsylvania (44), Oklahoma (18), Kentucky (16), and Wyoming (13). We decided to keep such inaccurate locations for our image acquisition but they can be easily found in the metadata (“accuracy” column) and excluded from the model training if desired. Also, the trained model can be utilized on such images to re-estimate the locations of the wells on them.

## Usage Notes

We collected extensive metadata, especially for positive examples. For each well location, we gathered multiple scene and display IDs along with the scene acquisition date and spatial resolution. This is sufficient to download several NAIP images from the Earth Explorer for each well location. This can be used to expand the current dataset with extra images or conduct change detection studies to understand environmental dynamics.

The tiles we obtained have a resolution of 512 × 512 pixels. However, users can generate tiles of other pixel resolutions. It is important to note that creating tiles with different pixel resolutions would necessitate acquiring the NAIP images from USGS. We supplied the necessary metadata and scripts, ensuring that users can redownload NAIP images for the specified well locations without needing to redo much of the effort here. Furthermore, if a buffer size other than 100 pixels is desired, the masks can be regenerated accordingly without downloading additional data from NAIP as metadata files contain pixel coordinates of the wells for each positive image.

There could be a concern about the balanced-frequency classes in our dataset, which do not accurately reflect the real-world scenario where abandoned wells are a minority. Users can choose any amount of negative examples for training model purposes. In addition, the model can be trained on images from particular states, as it could be easier to detect wells in states with less vegetation.

**Fig. 5:**
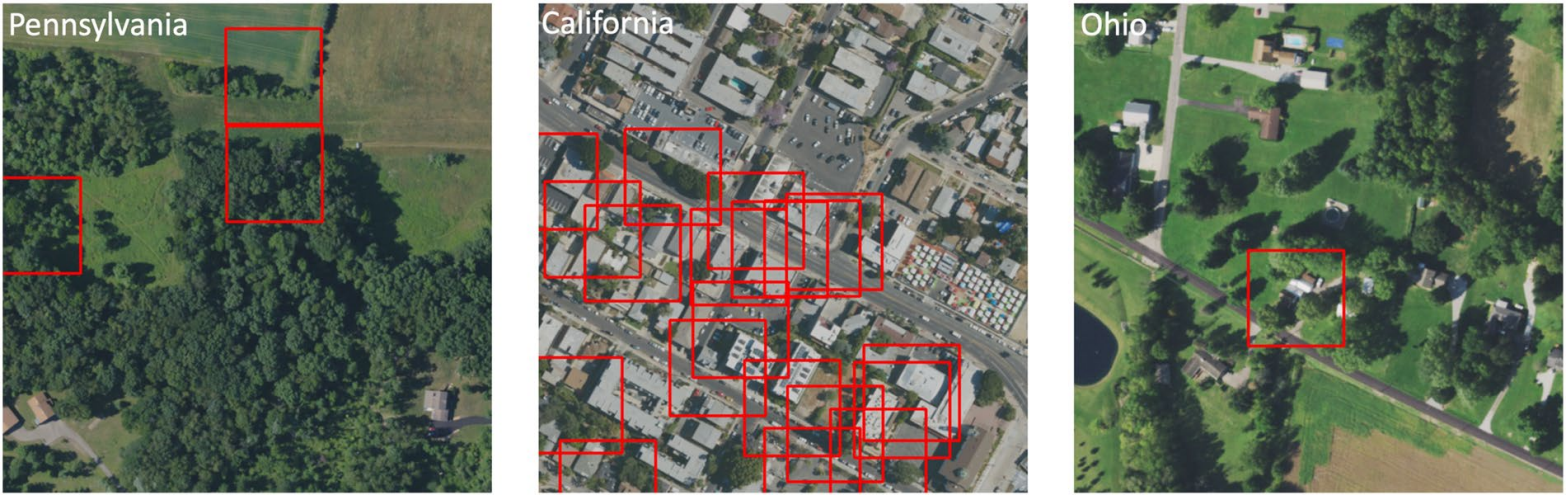
Several examples that we expect would prove challenging for a machine learning model are shown. Since many of these wells are quite old, visual information can be hard to find in aerial images, e.g., if they have become overgrown by vegetation (left), dense urban development (center), or less dense housing (right).

By scrolling through numerous images of oil and gas wells, we observed that a significant number of them are indeed hidden and cannot be detected by the naked eye, even with the use of high-resolution aerial imagery (Fig. 5). This challenge is further compounded by the presence of confounding objects such as certain rock formations or man-made structures, which could be mistaken for wells. Despite these complexities, we anticipate that trained machine learning models can effectively identify subtle changes in the surrounding areas that could indicate the presence of a well. For instance, the presence of a well pad, which is a cleared or constructed area used for drilling activities, may suggest a high likelihood of a nearby well. Additionally, access roads or pathways leading to a potential well site might also be detectable in the imagery. Vegetation in the vicinity of the wells can also offer clues; for example, vegetation health may be impacted, or its growth patterns may change due to methane emissions or other environmental effects associated with the well. Another potential indicator could be changes in soil color or texture that are often associated with drilling activities. We hope that machine learning models will learn to recognize these associated features, thereby improving our ability to identify wells that are not easily visible in aerial imagery.

Locating orphan oil and gas wells is a critical national and global need to clean up the mess left behind by our legacy of fossil fuel exploration. The number of wells is huge – at least hundreds of thousands, possibly millions and they are spread over large geographic regions. This is a classic problem of having many needles in a very large haystack. Methods are desperately needed to reduce the search space, so that previous resources like drones equipped with sensors can be used efficiently. By releasing this dataset, we aim to provide a resource that can spur further research in the field, particularly in support of the current government initiative to identify and locate orphaned wells, thereby contributing to environmental protection.

## Data Availability

The dataset is available at the Figshare repository^8^. The aerial imagery dataset (100 GB) provides a ready-to-use training dataset for deep learning models. This dataset is comprised of four folders which contain 512 × 512-pixel positive (58,882) and negative (62,066) images in TIFF format ("positive_images” and “neg_images” folders) and the corresponding segmentation masks in PNG format ("positive_masks” and “neg_masks” folders). Metadata related to the images is provided in two PKL file format data files (‘positive_df.pkl” and “negative_df.pkl”). The image characteristics metadata includes the name of each image, state, and latitude and longitude coordinates where the well is located, image spatial resolution, and pixel coordinates of the well on the image, as well as the spatial resolution and ID of the corresponding NAIP image. Additionally, we have collected a few alternative IDs for the NAIP images from different years and spatial resolutions for positive images.

## Code availability

Scripts used to obtain the dataset and metadata are available in the following GitHub repository: https://github.com/lanl/uow. Guidelines are provided in a README file that explains all instructions for their execution. Data is uploaded to the figshare repository.
